# Postnatal women’s perception on person-centered maternity care in twin cities of Rawalpindi and Islamabad: a descriptive study

**DOI:** 10.1186/s12884-023-05362-6

**Published:** 2023-01-21

**Authors:** Sumbal Hameed, Sheh Mureed, Rizwana Chaudhri, Shahzad Ali Khan, Mohsin Saeed Khan

**Affiliations:** 1 Health Services Academy, Islamabad, Pakistan; 2 Health Section, Ministry of Planning Development and Special Initiatives, Islamabad, Pakistan; 3 Obstetrics and Gynecology Department, Holy Family Hospital, Rawalpindi, Pakistan; 4 Vice Chancellor, Health Services Academy, Islamabad, Pakistan; 5 Department of Global Public Health, Karolinska Institute, Stockholm, Sweden

**Keywords:** Person-centered maternity care, Post-natal women, Respectful maternity care in Pakistan, Low middle-income countries

## Abstract

**Background:**

Person-Centered Maternity Care (PCMC) is known as one of the most important components of maternal care. Every woman has the ultimate right of respectful health care. Previous research documents that lack of supportive care and respectful behavior experienced by pregnant women can act as a barrier to the utilization of health care services. Few studies have used PCMC tool to document this phenomenon. The objective of this descriptive study was to assess the women’s perception of PCMC in Pakistan.

**Methods:**

Three hundred and seventy-seven (377) postnatal women of ages 18–49 years participated in the research. The study sites were secondary and tertiary care hospitals located in the twin cities of Rawalpindi and Islamabad. The PCMC tool used in this study is a validated scale with three sub-domains of i) communication and autonomy, ii) supportive care, and iii) dignity and respect. Data was analyzed using SPSS version 16, and descriptive and bivariate analysis was undertaken.

**Results:**

The PCMC mean score was 54 ± [10.7] out of 90. About half (55%) of women had good perception of PCMC. Sub-domain of supportive care scored the lowest as compared to the other two domains. Overall, 36% women reported physical abuse while 22% reported verbal abuse at the hands of the healthcare providers. Most of the women (88%) said that health providers did not introduce themselves. About 30% women claimed that health care providers never asked for permission before doing any medical procedures and 20% of women claimed that doctors did not describe the purpose of examination while 178 (47%) of women said that health provider explained the purpose of medications all the time, additionally, about 14% were never given the choice to ask questions.

**Conclusion:**

The study concluded that the majority of postnatal women perceived that they were not getting optimum Person-Centered Maternity Care. Some core aspects in supportive care domain were missing. In order to improve the quality of hospital-based childbirths, efforts are needed to improve the quality of care.

## Introduction

Improving maternal health and saving the lives of mothers remains at the center of global health and development initiatives [1]. According to World Health Organization (WHO), “Maternal Health is the health of a woman during pregnancy, childbirth and the postpartum period” [2]. According to Sustainable Development Goal (SDG) 3, by 2030, all signatory countries should have a Maternal Mortality Ratio (MMR) of less than 70 per 100,000 live births [3]. About 295,000 maternal deaths occurred globally in 2017, which were attributable to pregnancy related conditions and childbirth. Majority (94%) of maternal deaths were reported to have taken place in Low-Middle Income Countries (LMICs) [4]. However, most of the maternal deaths are preventable through skilled childbirth care and accessibility to emergency obstetric care services. According to the United Nations Children’s Fund (UNICEF,) from 2013 to 2018, approximately 76% of childbirths took place in hospitals [5]. Worldwide, in 2017, 80% of childbearing women attended at least one antenatal care (ANC) visit while 60% were served by skilled birth attendants, and 35% of women had access to postnatal care [6]. The increased usage of these maternity services resulted in a 29% decrease in maternal deaths from 390,185 in 2000 to 275,288 in 2015 [7]. In order to further reduce the global burden of maternal deaths and maternal morbidity, universal access to high quality, safe and respectful maternal care has been cited as a proactive intervention [8, 9].

Quality of care is “an efficient, effective, equitable, timely, safe and person-centered care provided to the patients” [10]. Quality of care in maternal health refers to the degree to which maternal health services for individuals and populations increase the likelihood of timely and appropriate treatment to achieve desired outcomes that are both consistent with current professional knowledge and upholding fundamental reproductive rights [11]. Quality of care encompasses both the provision of care and experience of care. Provision of care is the practice provided to people for their routine care check-ups and management of problems and/or complications. Experience of Care, on the other hand, is a person-centered approach that includes preservation of dignity & respect, supportive care and effective communication [10]. The quality of service experience enhances patient’s knowledge and satisfaction level leading to improved health outcomes. Quality of care thus plays an important role in reducing burden of disease and cost of treatment [12].

Person-Centered Maternity Care (PCMC) is a critical component of the experience of care. PCMC refers to “care provided to women during childbirth that is respectful and responsive to individual woman and their families’ preferences, needs, and values” [13]. PCMC highlights respectful maternity care as a broader part of women-centered care. Due to poor PCMC, women are discouraged from using hospital-based maternal health facilities and giving birth in the health facilities, which contributes directly and indirectly to adverse and poor pregnancy outcomes [14, 15]. On the other hand, PCMC helps in identifying and addressing disrespect, abuse and mistreatment [16].

Qualitative research studies on the experience of care have focused on disrespect and abusive behaviors towards women. Several quantitative studies are published on disrespect and abuse which provide different estimates using different methodological approaches in different settings. A study in Kenya revealed 20% prevalence of disrespect and abuse based on only question, that is by asking women whether at any point during delivery, they were treated disrespectfully by health care providers [17]. Similar studies in different settings used Browser and Hills framework to document the prevalence of disrespectful care [18–22]. A study in Tanzania reported the prevalence of disrespect and abuse being as high as 15% [18]. Two studies from India reported 21 and 77% disrespectful behavior based on different measures [19, 20]. Studies conducted in Peru and Nigeria reported over 90% prevalence of disrespect and abuse [21, 22]. Studies conducted in Sub-Saharan Africa measured the prevalence of non-consented care from below 1% in some research studies while other reported more than 20% [13].

According to the Pakistan’s Maternal Mortality Survey 2019, the maternal mortality ratio is 186 maternal deaths per 100,000 live births. This has been attributed to low utilization of health services and other factors [23]. The results of Pakistan Demographic and Health Survey 2017–18 shows that the proportion of births managed by skilled birth attendants is about 69%. The proportion of women with 4 or more antenatal care visits was 51% in 2017–18. The proportion of women with a postnatal check-up within 2 days after delivery has remained largely unchanged between 2012 and 13 (61%) and 2017–18 (62%) [24]. Studies from Pakistan have reported more than 90% disrespect and abuse to pregnant women, but these studies used different scales to measure respectful care [25]. To further reduce the burden of preventable maternal deaths in Pakistan, the quality of maternity care must be optimized.

The objective of this research was to assess post-natal women’s perception of PCMC in three government hospitals in the twin cities of Rawalpindi and Islamabad. In this study, PCMC scale was used, which is a reliable and validated questionnaire tested and developed [26] in a context similar to that of Pakistan.

## Method

A cross-sectional descriptive study was conducted in three government-operated public hospitals (one tertiary and two secondary care hospitals) in the twin cities of Rawalpindi and Islamabad during June–August 2019.

The study population for this research were post-natal women who had recently given birth in one of these public hospitals. The inclusion criteria for the study population were as follows: women aged 18–49 years old; who had given live birth, either vaginally or through C-section; who were within the post-natal period up to six-weeks after birth. Women who were referred from other hospitals but gave birth in the selected hospitals were also included. Women who suffered from any complication, during any stage of pregnancy such as puerperal sepsis, intrauterine death or miscarriage, eclampsia, PPH were excluded. Women diagnosed with mental health issues such as depression, anxiety or post-natal depression were also excluded. Afghan refugees or other nationals were also not included in this study.

Sample size was calculated by using an online sample size calculator for population proportion, [27] with a 0.05 margin of error, confidence interval of 95%, and an expected prevalence of 57% of respectful care (based on previous research in Pakistan), a sample size of 377 was required [28]. The consecutive sampling method was employed to enroll the women. The principal investigator and a trained research assistant collected the data. After taking a written informed consent from participants, the interviews were conducted in the post-natal wards. Data was collected on paper in Urdu language. The PCMC questionnaire was translated by the principal investigator from English into Urdu (National language), and then back translated from Urdu to English by the second co-author. After comparing both versions to assess discrepancies, and after developing consensus, the final version was translated into Urdu for data collection.

The study tool was the PCMC scale first developed and validated in Kenya, followed by validation in India [26, 29]. The PCMC scale aims to capture and present quantitatively all aspects of Respectful Maternity Care as prescribed in WHO Quality of Care framework. This tool was developed using standard procedures such as: literature reviews to define the construct of PCMC and identification of its sub-domains; expert reviews were conducted to assess content validity; cognitive interviews were conducted to evaluate the clarity, wording, and appropriateness of items. The PCMC questionnaire was found to have high content validity, offered good internal consistency, and high reliability with Cronbach Alpha value to be more than 0.8, while for the sub-domains the value ranged between acceptable levels (0.6 and 0.8) in multiple studies [26, 29]. The justification for selecting this scale is that, it was developed using standard protocols, and more importantly it was validated in similar socio-economic and health system settings analogous to Pakistan.

The PCMC scale consists of 30 items with three key sub-domains: i) dignity and respect (D&R), ii) communication and autonomy (C&A), and iii) supportive care (SC). Each item of the questionnaire consists of four-point response, each on the scale of “0 to 3” such as: 0 (No, never), 1 (Yes, a few times), 2 (Yes, most of the time), and 3 is (Yes, all the time). The overall PCMC score is an additive score computed by adding individual responses of the 30-items PCMC statements/questions and has range of score from zero to 90. Mean ± SD score was calculated for the additive scores, and women were categorized into high and low PCMC groups based on the mean cutoff value. The C&A sub-domain consisted of 9 items and its score ranged from zero to 27. The D&R sub-domain with 6 items had a score ranged from zero to 18. The SC sub-domain had 15 items and its score ranged from zero to 45. The questions addressing verbal and physical abuse were reverse coded so that high numbers represent good care. The other variables in the study tool included age, parity, household monthly income, education, and employment status, number of ante-natal visits and reproductive history to capture the provider and facility characteristics.

Data was processed and analyzed using SPSS V. 16 (SPSS Inc., Chicago Illinois, USA). Frequencies, proportions, mean, standard deviation, minimum and maximum were calculated for the descriptive data. A Pearson’s chi-square was employed to assess factors associated with overall PCMC with significance level at p = < 0.05.

Ethical clearance was given by Health Services Academy and the public sector health facilities, where the survey was conducted. All participants were informed about the objective of the study and what was expected of them. They also were informed that the data would be kept anonymous and that they could refuse to participate in the interview at any given time without affecting their future interaction with the service providers at the health facilities.

## Result

The questionnaire was administered to total of 389 women who recently had given birth in health facility, out of which 12 women didn’t finish the interview due to various reasons. The average age of the respondents was 27 years. The median parity was 2 children per woman ranging from 1 to 7. Majority 36% (*n* = 137) of the women had completed ten years of schooling. Ninety-six percent (*n* = 363) of the women were housewives and were not employed in any formal sector. About 33% of the women reported having a household monthly income between US $61–122 (conversion rate 1US$ = 163 Pak Rupee). Among the 377 women who completed the interviews, 63% (*n* = 236) had more than 4 ante-natal visits. Ninety-three percent women reported having their children delivered by female doctors. Details are given in Table 1.

**Table 1:** Demographic characteristics and past pregnancy history of post-natal women (*n* = 377)

S.No.	Variables	Responses	Frequency	Percentage
1.	Age Distribution	Less than or equal to 24	116	31%
		25–35	249	66%
	Mean Age	Greater than 35	12	3%
		26.8 years		
2.	Parity Median	2 children per woman		
3.	Employment	Private	9	3%
		Government	5	1%
		Housewife	363	96%
4.	Education	Uneducated	63	17%
		Up to Fifth grade	104	28%
		Up to Tenth grade	137	36%
		Up to twelfth grade	73	19%
5.	Monthly Household income in US Dollars	30$-60$	104	28%
		61$-122$	123	33%
		123$-184$	92	24%
		Above 184$	58	15%
6.	Ante-natal visits	None	5	1%
		Less than 4	60	16%
		4	76	20%
		More than 4	236	63%
7.	Delivery provider	Nurse	25	6.7%
		Midwife	1	0.3%
		Doctor	351	93%

In the sub-domain of Dignity and Respect (D&C), about 51% (*n* = 194) of the women perceived that they were treated with respect all the time. During their time at the facility, about 36% (n = 137) of the women reported experiencing physical abuse once or more. However, verbal abuse by health care provider was reported less by only 22% women. About half (47%) of the women observed that most of the time, their information was kept confidential in the health facility.

In the sub-domain of Communication and Autonomy (C&A), majority (88%) of the women reported that the health care providers never introduced themselves. About two-third (66%) of the women recalled that the health care providers called them by their names all the time. Almost half (43%) of the women felt that the health providers involved them in their care all the time. More than two-thirds (70%) of the women said that health care providers asked for permission before conducting physical examination on them. Majority 80% of the women said that the health providers explained the purpose of medication, procedures and examination being administrated.

Regarding perception of women in the Supportive Care (S&C) sub-domain, almost half of the women (42%) reported that they had to wait very long to receive care. Majority of the women (93%) said that that no one was allowed to stay with them during delivery. However, about the same majority (92%) of the women said that that they didn’t want any one accompanying them during delivery. More than half of the women (58%) perceived that health facility was too crowded ranging from sometimes to all times. It was reported by many women (67%) that the environment and washrooms of the health facility were clean. Regarding basic utilities of the facility, women observed that electricity and water was available all the time 85 and 89% respectively. Only 60% of the women said that they felt safe in the health facility. Details of responses to PCMC items by sub-domain are given in Table 2.

**Table 2:** Responses to PCMC scale (*n* = 377)

S.NO.	Questions	Responses	N (%)
**Dignity and Respect sub-domain**			
1.	Did the health providers at the facility treat you with respect?	No, never	15 (4%)
		Yes, a few times	83 (22%)
		Yes, most of the time	85 (23%)
		Yes, all the time	194 (51%)
2.	Did the doctors, nurses, and other staff at the facility treat you in a friendly manner?	No, never	16 (4%)
		Yes, a few times	87 (23%)
		Yes, most of the time	93 (25%)
		Yes, all the time	181 (48%)
3.	Did you feel the health providers shouted at you, scolded, insulted, threatened, or talked to you rudely?*	No	292 (78%)
		Yes, once	57 (15%)
		Yes, a few times	24 (6%)
		Yes, many times	4 (1%)
4.	Did you feel like you were treated roughly like pushed, beaten, slapped, pinched, physically restrained, or gagged?*	No	240 (64%)
		Yes, once	57 (15%)
		Yes, a few times	48 (13%)
		Yes, many times	32 (8%)
5.	During examinations in the labour room, were you covered up?	No	85 (23%)
		Yes, a few times	133 (35%)
		Yes, most of the time	116 (31%)
		Yes, all the time	43 (11%)
6.	Do you feel like your health information was or will be kept confidential at this facility?	No	4 (1%)
		Yes, a few times	33 (9%)
		Yes, most of the time	177 (47%)
		Yes, all the time	163 (43%)
**Communication and Autonomy sub-domain**			
7.	During your time in the health facility did the health providers introduce themselves to you when they first came to see you?	No, none of them	330 (88%)
		Yes, a few of them	28 (7%)
		Yes, most of them	18 (5%)
		Yes, all of them	1 (0.3%)
8.	Did the health providers call you by your name?	No	16 (4%)
		Yes, a few times	35 (9%)
		Yes, most of the time	77 (21%)
		Yes, all the time	249 (66%)
9.	Did you feel like the health providers at the facility involved you in decisions about your care?	No	77 (20%)
		Yes, a few times	45 (12%)
		Yes, most of the time	94 (25%)
		Yes, all the time	161 (43%)
10.	During the delivery, do you feel like you were able to be in the position of your choice?	No	131 (35%)
		Yes, for a short time	121 (32%)
		Yes, most of the time	78 (21%)
		Yes, all the time	47 (12%)
11.	Did the health providers at the facility speak to you in a language you could understand?	No	9 (2%)
		Yes, a few times	51 (14%)
		Yes, most of the time	79 (21%)
		Yes, all the time	238 (63%)
12.	Did the health providers at the facility ask your permission or consent before doing procedures on you?	No	111 (30%)
		Yes, a few times	129 (34%)
		Yes, most of the time	84 (22%)
		Yes, all the time	53 (14%)
13.	Did the health providers explain to you why they were doing examinations or procedures on you	No	104 (28%)
		Yes, a few times	137 (36%)
		Yes, most of the time	83 (22%)
		Yes, all the time	53 (14%)
14.	Did the health providers explain to you why they were giving you any medicine?	No	75 (20%)
		Yes, a few times	51 (13%)
		Yes, most of the time	73 (19%)
		Yes, all the time	178 (47%)
15.	Did you feel you could ask the health providers at the facility any questions you had?	No	54 (14%)
		Yes, a few times	58 (16%)
		Yes, most of the time	95 (25%)
		Yes, all the time	170 (45%)
**Supportive Care sub-domain**			
16.	How did you feel about the amount of time you waited? Would you say it was?*	Very short	56 (15%)
		Somewhat short	73 (19%)
		Somewhat long	89 (24%)
		Very long	159 (42%)
17.	Did the health providers at the facility talk to you about how you were feeling?	No	71 (19%)
		Yes, a few times	55 (15%)
		Yes, most of the time	118 (31%)
		Yes, all the tim	133 (35%)
18.	Did the health providers at the facility try to understand your anxieties?	No	44 (12%)
		Yes, a few times	87 (23%)
		Yes, most of the time	115 (30%)
		Yes, all the time	131 (35%)
19.	When you needed help, did you feel the health providers at the facility paid attention?	No	42 (11%)
		Yes, a few times	70 (19%)
		Yes, most of the time	105 (28%)
		Yes, all the time	160 (42%)
20.	Do you feel the health providers did everything they could to help control your pain?	No	27 (7%)
		Yes, a few times	56 (15%)
		Yes, most of the time	113 (30%)
		Yes, all the time	181 (48%)
21.	Were you allowed to have someone you wanted (outside of staff at the facility, such as family or friends) to stay with you during labour?	No	351 (93%)
		Yes, a few	17 (4%)
		Yes, most of the time	6 (2%)
		Yes, all the time	3 (1%)
22.	Were you allowed to have someone you wanted to stay with you during delivery?	No	347 (92%)
		Yes, a few times	20 (5%)
		Yes, most of the time	7 (2%)
		Yes, all the time	3 (1%)
23.	Did you feel the health providers at the facility took the best care of you?	No	31 (8%)
		Yes, a few times	68 (18%)
		Yes, most of the time	83 (22%)
		Yes, all the time	195 (52%)
24.	Did you feel you could completely trust the health providers at the facility with regards to your care?	No	41 (11%)
		Yes, a few times	49 (13%)
		Yes, most of the time	85 (22%)
		Yes, all the time	202 (54%)
25.	Do you think there were enough health staff in the facility to care for you?	No	95 (25%)
		Yes, a few times	59 (15%)
		Yes, most of the time	62 (17%)
		Yes, all the time	161 (43%)
26.	Thinking about the labour and postnatal wards, did you feel the health facility was crowded?*	No	158 (42%)
		Yes, a few times	95 (25%)
		Yes, most of the time	49 (13%)
		Yes, all the time	75 (20%)
27.	Thinking about the wards, washrooms, and the general environment of the health facility, would you say the facility was very clean, clean, dirty, or very dirty?	Very dirty	25 (7%)
		Dirty	88 (23%)
		Clean	253 (67%)
		Very clean	11 (3%)
28.	Was there water in the facility?	No	11 (3%)
		Yes, a few times	16 (4%)
		Yes, most of the time	31 (8%)
		Yes, all the time	319 (85%)
29.	Was there electricity in the facility?	No	4 (1%)
		Yes, a few times	10 (3%)
		Yes, most of the time	26 (7%)
		Yes, all the time	337 (89%)
30.	In general, did you feel safe in the health facility?	No	24 (7%)
		Yes, a few times	65 (17%)
		Yes, most of the time	61 (16%)
		Yes, all the time	227 (60%)

(Note: * = negative statement)

Against the best score of 90, the average PCMC score in this study was 54. The average score of D&R sub-domain was 13 out of 18, for C&A sub-domain score was 14 out of 27, and 27 out of 45 score for the S&C sub-domain. Table 3 shows results of overall scores of PCMC and its sub-domains.

**Table 3:** Distribution of overall PCMC and its Sub-domains

PCMC (*n* = 377)	Mean	[SD; min-max]
Overall PCMC scale	54	[10.7; 14–80]
Dignity & Respect	13	[3.2; 3–18]
Communication & Autonomy	14	[4.1; 4–25]
Supportive Care	27	[6.2; 4–41]

The 30-item PCMC scale has range of score from (0–90), each on the scale of “0 to 3”. The C&A sub-domain consists of 9 items and score range from (0–27). The D&R sub-domain consists of 6 items and score range from (0 to 18). The SC sub-domain has 15 items, and scores range from (0–45).

**Fig. 1:**
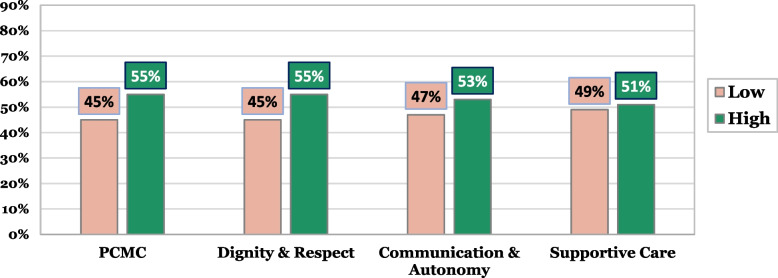
Distribution of PCMC score by its sub-domains

Based on the mean score, the overall PCMC and sub-domains were converted and categorized into high and low respectful maternity care. Overall, 55% of the women perceived that they received high quality PCMC at these health facilities. For the different sub-domains of PCMC, it was found that 55% women graded dignity and respect as “high”, 53% evaluated communication and autonomy to be “high” level and 51% of the women perceived “high” supportive care. Fig. 1 shows distribution of PCMC score by its sub-domains.

Cross-tabulation between socio-demographic and reproductive factors was done to assess factors associated with PCMC. Unfortunately, none of the factors were found to be statistically significant. Details are given in Table 4.

**Table 4:** Cross-Tabulation between Socio-Demographic and Reproductive factors with PCMC using Chi-square test (*n* = 377)

S. NO.	Variable	Categories	PCMC		***p***-value
			Low	High	
			N (%)	N (%)	
**1.**	**Age group**				0.137
		Less than or equal to 24 years	53 (46%)	63 (54%)	
		25–35 years	114 (46%)	135 (54%)	
		Greater than 35	2 (17%)	10 (83%)	
**2.**	**Employment**				0.225
		Private	6 (67%)	3 (33%)	
		Government	1 (20%)	4 (80%)	
		Housewife	162 (45%)	201 (55%)	
**3.**	**Education**				0.422
		Uneducated	29 (46%)	34 (54%)	
		Up to fifth grade	41 (39%)	63 (61%)	
		Up to Tenth grade	61 (45%)	76 (55%)	
		Up to twelfth grade	38 (52%)	35 (48%)	
**4.**	**Monthly Income in USD**				0.080
		30–60 $	44 (42%)	60 (58%)	
		61–122 $	46 (37%)	77 (63%)	
		123–184 $	49 (53%)	43 (47%)	
		Above 184 $	30 (52%)	28 (48%)	
**5.**	**Antenatal visits**				0.787
		None	2 (40%)	3 (60%)	
		More than 4	103 (44%)	133 (56%)	
		Less than 4	26 (43%)	34 (57%)	
		Only 4	38 (50%)	38 (50%)	
**6.**	**Delivery provider**				0.633
		Nurse	12 (48%)	13 (52%)	
		Midwife	0 (0%)	1 (100%)	
		Doctor	157 (45%)	194 (55%)	

(Note: Significance level at p = < 0.05; USD = United States Dollar)

## Discussion

This study explored women’s perception of person-centered maternity care using PCMC scale in the twin cities of Rawalpindi and Islamabad. To the best of our knowledge, this is the first study in Pakistan to use PCMC scale to explore this phenomenon. This study provides a starting point to assess the situation of respectful maternity care in Pakistan as an important component for improving quality of care during pregnancy and childbirth. Our study found that the overall PCMC mean score was 54 out of the maximum score of 90, which indicates a moderate level of patient-centered care experienced by women. This finding is similar to other countries, such as Kenya, Ghana and India, where it was also found that about half of the women were not getting optimum PCMC during childbirth across four different study settings [13].

Findings from dignity and respect sub-domain suggest that approximately 36% of the women reported physical abuse once or more and verbal abuse was reported by 22% women only. However, a study in Pakistan reported 35% verbal abuse and 15% physical abuse [30]. This may be because of the study setting since due to high patient load in government hospitals, women may have been verbally/physically mishandled. Many previous researches have used different methodological approaches to capture disrespectful care and have reported varying prevalence between 15 to 98% [31].

The score for the communication and autonomy sub-domain was also sub-optimal with only 53% perceiving high level of the same. Most of our study’s women (88%) in our study reported that the health care providers never introduced themselves. This finding is consistent with Ghana and Urban Kenya, where 87 and 85% of women reported that the health care providers never introduced themselves respectively [13]. Our study suggests that 30% reported non-consented care, whereas a study in Pakistan reported 81% non-consented care. Due to high patient inflow, a low doctor-to-patient ratio, and a lack of training on person-centered care, this important ethical element is not being performed by the health care providers.

The score for the supportive care sub-domain turned out to be the lowest out of the three. This result is also consistent with previous studies on PCMC. In earlier studies [ (13)] between 24 to 31% of the women reported that the providers talked to them all the time, about how they felt. This is consistent with our findings; 35% women were allowed to share their feelings. The results from an analysis of a similar study in Rural Kenya [13] showed that approximately 52% of the women felt that the facility provided high quality care, which is also consistent with the results of this study. Findings from past studies suggest that more than 70% of the women felt safe at the facility all the time, and our study showed that approximately 60% of the women felt safe in the facility.

Experience of Care is an essential part of quality of maternity care. Therefore, it should be incorporated in all health policies. Respectful care depends not only on the behaviour of health providers but also on the health facility infrastructure. Health care services are provided to the women with dignity and respect in this conducive environment. Therefore, efforts to improve respectful care should focus on the health system, structure of health facilities, provision of services, attitude and behavior of health care providers that generate mistreatment and disrespectful care. In addition, person-centered respectful maternal health services should be monitored at community, primary and others levels in Pakistan. Our study indubitably demonstrates that too much work is needed in Pakistan to improve the quality and experience of facility based maternal care services (that patients receive during delivery in health facilities).

A limitation of our study was the sampling design, where consecutive sampling was used, hence the external validity of the results is questionable. As this study was conducted within the health facility, the women might have felt hesitant to answer all questions sincerely, which could lead to under-reporting. However, this was considered during the study design. The data collectors were trained to build trust and ensure that this would not affect their care. Twelve women started and did not finish the questionnaire due to various reasons. As this study was conducted in three hospitals located in urban settings, hence findings cannot be generalized to other settings such as rural, private sector, primary level settings.

There are many strengths of this study. First, the data collection tool is a validated, and reliable tool developed and tested in similar setting. Second, this PCMC tool has both objective and subjective questions and encapsulates many contextual and individual-level factors. Third, the hospitals selected for this study represents the urban public-sector hospitals in the country, meaning that identified issues can be used to improve PCMC. Finally, recall bias is lower in our study because the respondents were interviewed within six weeks of giving birth.

## Conclusion

Approximately half (55%) of the women perceived that they were receiving high quality PCMC in the health facilities. Out of the three sub-domains, the core aspects of Supportive Care were found to be deficient. To improve PCMC, trainings and reinforcements are needed to incorporate accountability and measurement mechanisms. Refresher trainings should be provided to the health care providers about the rights of providers and the patients. Future studies should use findings from this study to develop and test health system interventions to improve person-centered respectful care in similar settings. A mixed method approach may be employed to assess health care providers’ perceptions on the same phenomena.

## Data Availability

The dataset supporting to this article are available upon request to the author.

## Abbreviations

PCMC: Person Centered Maternity Care
QoC: Quality of Care
RMC: Respectful Maternity Care
D&R: Dignity and Respect
C&A: Communication and Autonomy
SC: Supportive Care
LMICs: Low Middle-Income Countries.
